# Recognizing Pelvic Venous Disease in Chronic Pelvic Pain: A Primary Care Case Report

**DOI:** 10.7759/cureus.117767

**Published:** 2026-10-11

**Authors:** Sandra Rodrigues, Francisco Dias

**Affiliations:** 1 São Vicente Family Health Unit, Unidade Local de Saúde do Tâmega e Sousa, Penafiel, PRT; 2 União Penafidelis Family Health Unit, Unidade Local de Saúde do Tâmega e Sousa, Penafiel, PRT

**Keywords:** chronic pelvic pain, pelvic congestion syndrome, pelvic varices, pelvic venous system, primary health care

## Abstract

Chronic pelvic pain is a common and challenging condition with a broad differential diagnosis and a substantial impact on quality of life. Pelvic congestion syndrome (PCS) is a clinical manifestation of pelvic venous disease that may involve venous reflux, obstruction, collateralization, and dilation across different pelvic venous territories. Its non-specific clinical presentation and overlap with gynecological, gastrointestinal, and musculoskeletal disorders may lead to significant diagnostic delay. A 42-year-old woman presented to a general and family medicine consultation with a six-year history of chronic pelvic pain, progressively worsening over the previous six months. She described persistent pressure-like pelvic pain, rated 7/10 in intensity, aggravated by prolonged standing and associated with severe deep dyspareunia, which prevented sexual intercourse, associated with low back pain radiating to the pelvis and both thighs, lower-limb heaviness, and significant sleep disturbance. She denied urinary symptoms or a relationship with the menstrual cycle. During the previous six years, she had undergone repeated medical assessments, with symptoms being attributed to recurrent gynecological infections, probable irritable bowel syndrome, and other non-specific causes. Previous investigations, including colonoscopy and emergency department assessments, had not identified a clear etiology for her symptoms. Physical examination revealed hypogastric tenderness and mild superficial lower-limb varicosities, without vulvar or perineal varices. Pelvic and lumbar computed tomography (CT) demonstrated bilateral dilated periuterine veins measuring up to 13 mm, raising suspicion for a symptomatic pelvic venous disorder. The patient was referred to vascular surgery for further assessment; however, specialist evaluation remained pending at the time of manuscript revision. Pelvic venous disease should be considered in women with persistent pelvic pain, particularly when symptoms worsen with prolonged standing or sexual intercourse and are accompanied by low back pain or lower-limb heaviness. The absence of vulvar varices or previous inconclusive investigations does not exclude a possible pelvic venous etiology. This case illustrates how the non-specific presentation of pelvic venous disease may result in years of fragmented investigation and delayed recognition. Continuity of care in general and family medicine facilitated a longitudinal review of the patient's symptoms and previous investigations, allowing recognition of a pattern suggestive of pelvic venous disease. Greater awareness of pelvic venous disease in primary care may facilitate earlier consideration and appropriate specialist referral in women with otherwise unexplained chronic pelvic pain.

## Introduction

Chronic pelvic pain (CPP) is generally defined as persistent or recurrent pain perceived in structures related to the pelvis lasting for at least three months, although a threshold of six months has traditionally been used in some gynecological definitions [1]. It may involve the lower abdomen, lumbosacral region, or perineum and can have substantial functional, emotional, and quality-of-life consequences. It affects between 6% and 27% of women aged 18 to 50 years [2,3] and is frequently associated with anxiety, depression, sleep disturbances, and sexual dysfunction. CPP is often multifactorial and may involve nociceptive, neuropathic, and nociplastic pain mechanisms, including central sensitization, as well as psychosocial factors.

Among the causes of CPP is pelvic congestion syndrome (PCS), which is responsible for up to 30% of cases [2]. Pelvic venous disease may involve venous reflux, obstruction, collateralization, and dilatation across different pelvic venous territories, with PCS representing one of its possible clinical manifestations [3,4]. Although potentially treatable, it remains underdiagnosed due to the non-specific nature of its clinical presentation and the limited awareness of health professionals.

## Case presentation

A 42-year-old woman, a textile industry worker, presented in a general and family medicine consultation with worsening chronic pelvic pain of six years' duration, associated with low back pain, severe deep dyspareunia, and heaviness of the lower limbs. Her personal history included general anxiety disorder and depression. Her obstetric history was significant for one uncomplicated term vaginal delivery (G1P1). Her daily medication was a progestogen-only contraceptive.

She reported chronic low back pain that had worsened over the previous six months, radiating to the pelvis and both thighs, associated with lower-limb heaviness and a subjective sensation of intermittent weakness. No objective loss of muscle strength or sensory deficits in the lower limbs were identified on neurological examination. She denied urinary or fecal retention or incontinence and saddle anesthesia. Concurrently, she presented with generalized, persistent, pressure-like pelvic pain, rated 7/10 in intensity, aggravated by prolonged standing and associated with severe deep dyspareunia, which prevented sexual intercourse and had a significant impact on sleep. The deep dyspareunia was not associated with post-coital aching. She reported occasional vulvar fullness and mild superficial lower-limb varicosities. She denied premenstrual worsening, dysmenorrhea, urinary urgency, abnormal uterine bleeding, previous deep vein thrombosis, pelvic surgery, or a history of, or previous suspicion of, endometriosis.

The patient had undergone repeated assessments over several years. In 2020, a total colonoscopy for abdominal pain, distension, and constipation was normal, and irritable bowel syndrome was subsequently considered. In 2021, persistent abdominal, pelvic, and low back pain prompted further investigation; blood tests, abdominal and gynecological ultrasonography, and lumbosacral radiography showed no relevant abnormalities. Gastroenterology assessment was subsequently pursued. During 2022-2023, recurrent genital pain and vaginal discharge prompted further evaluation; vaginal microbiological testing was negative, and repeat gynecological ultrasonography was normal. In April 2026, worsening pelvic and low back pain, deep dyspareunia, and lower-limb heaviness prompted renewed investigation. Lumbar radiography and urinalysis performed during an emergency department visit were unremarkable, as were subsequent repeat abdominal and gynecological ultrasonography and lumbosacral radiography. Persistent symptoms ultimately prompted abdominopelvic and lumbar computed tomography (CT).

On physical examination, the abdomen was soft and depressible, with hypogastric tenderness and no signs of peritoneal irritation. Bimanual pelvic examination was unremarkable, with no cervical motion tenderness, adnexal tenderness, or palpable adnexal masses. Straight-leg raising (Lasègue) was negative, and neurological examination of the lower limbs showed no objective motor weakness or sensory deficits. Mild superficial lower-limb varicosities were present, without peripheral edema. No vulvar or perineal varices were identified.

Non-contrast abdominopelvic and lumbar CT demonstrated bilateral dilated periuterine veins measuring up to approximately 13 mm, raising suspicion for a symptomatic pelvic venous disorder (Figures 1-3). The radiology report did not provide measurements of the ovarian veins or information regarding gonadal venous reflux, internal iliac venous involvement, or left renal or iliac vein compression. Additional findings included a small fat-containing umbilical hernia (7 × 9 mm) and, on lumbar CT, grade I L4 anterolisthesis, mild dextroconvex scoliosis, and degenerative spondylodiscal changes without evidence of neural compression.

**Figure 1:**
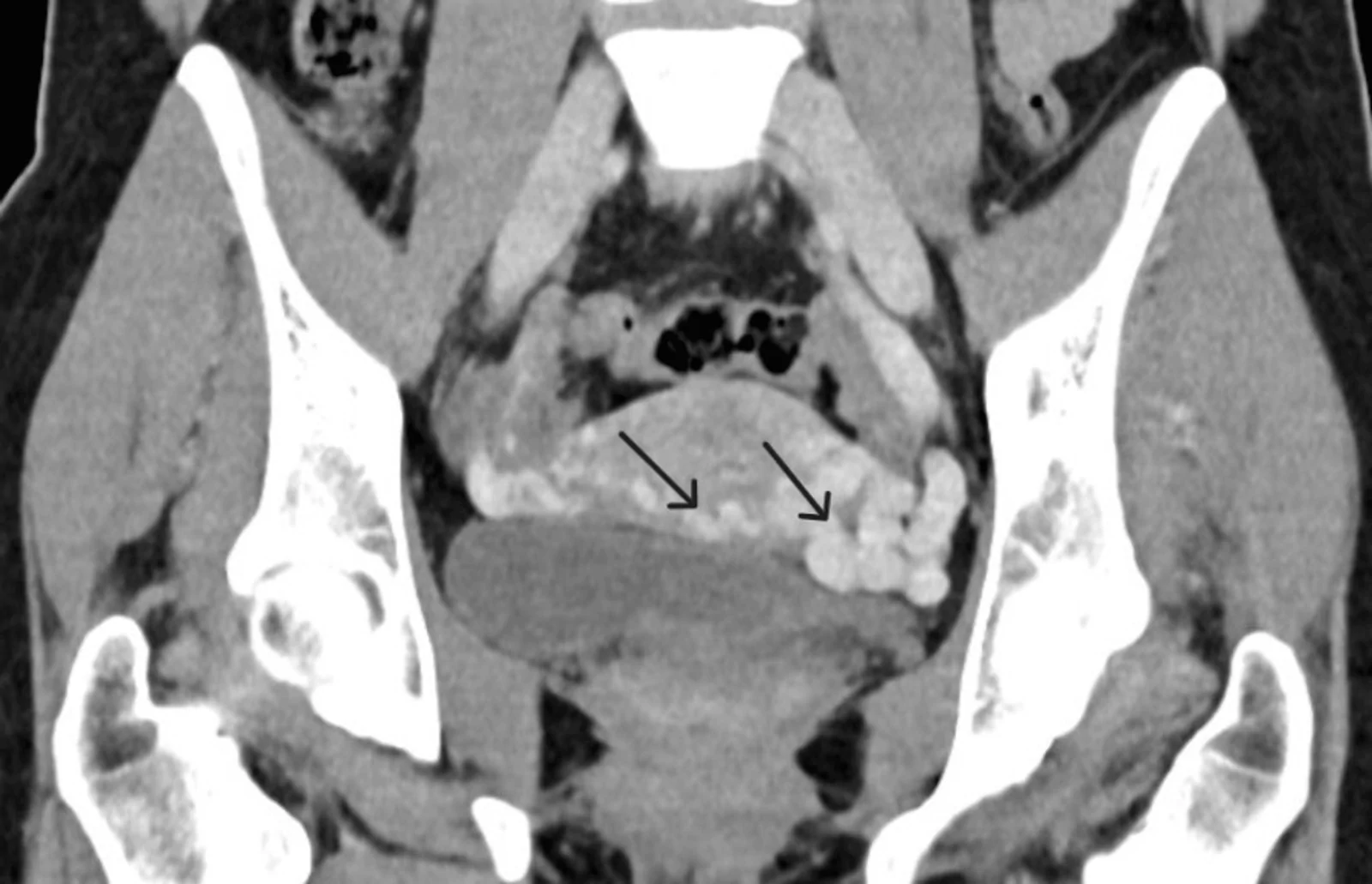
Pelvic CT scan (coronal view) showing bilaterally dilated periuterine veins (arrows)

**Figure 2:**
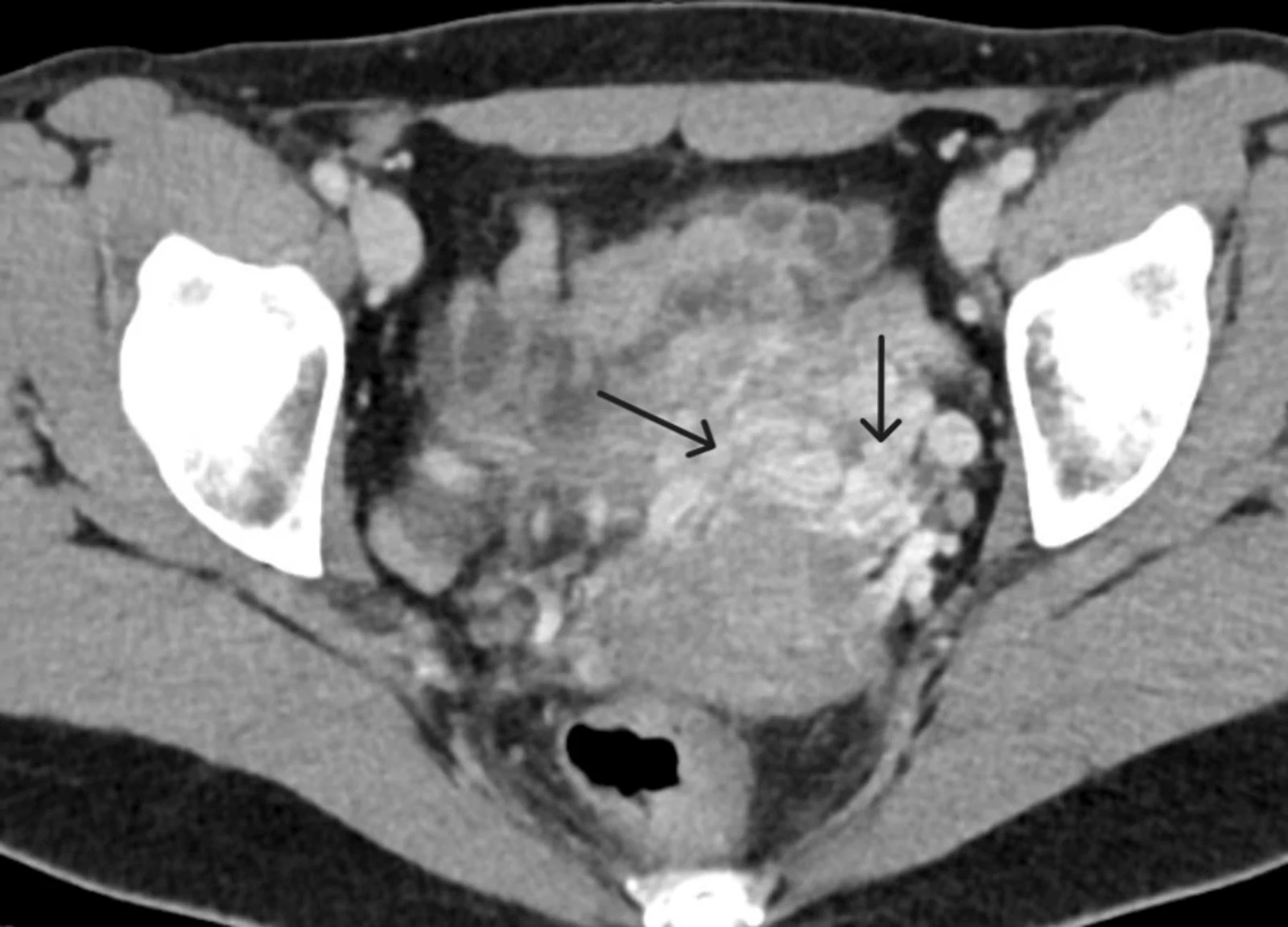
Pelvic CT scan (axial view) showing bilaterally dilated periuterine veins (arrows).

**Figure 3:**
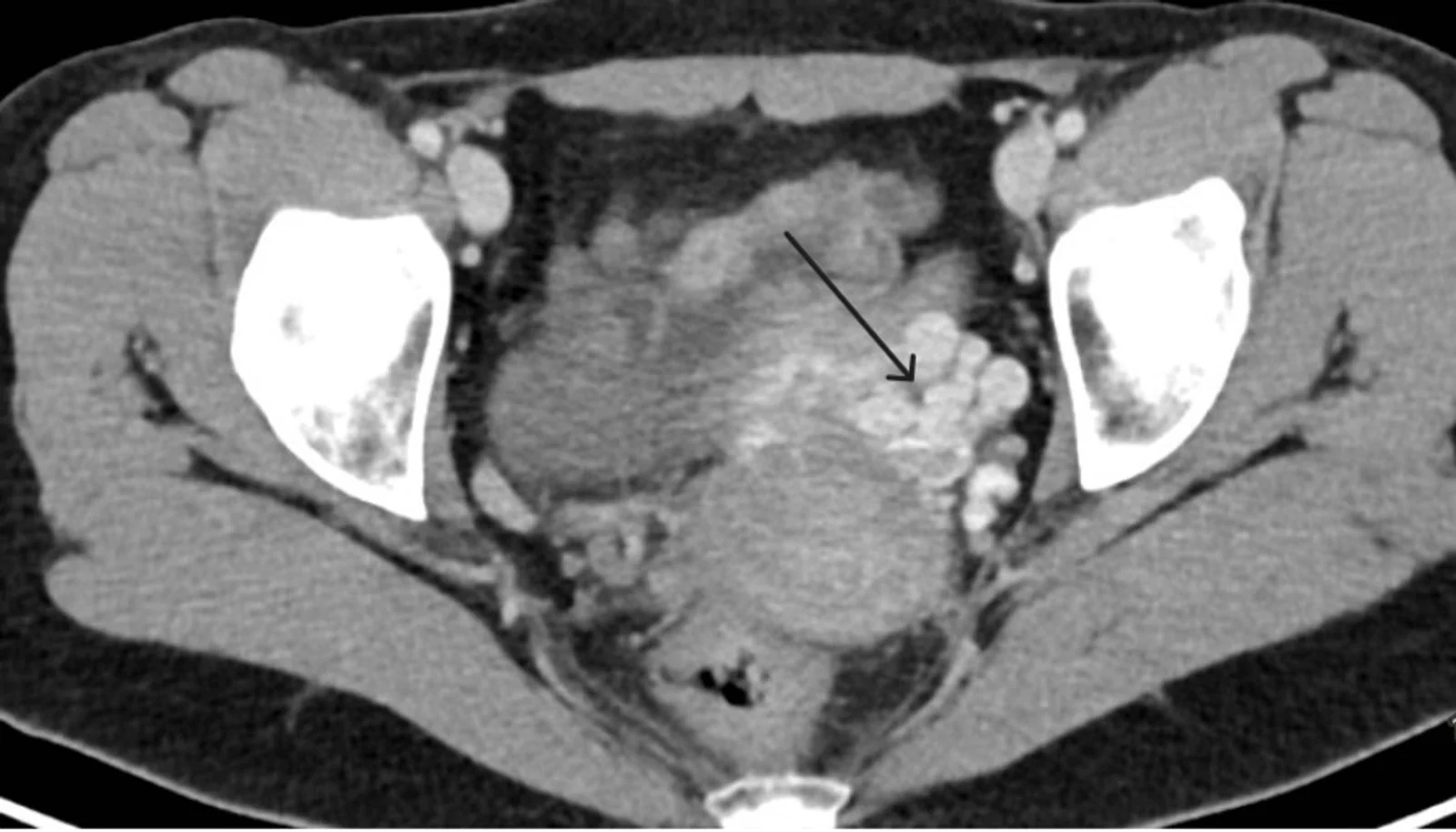
Pelvic CT scan (axial view) showing bilaterally dilated periuterine veins (arrow).

The patient was referred to vascular surgery for further assessment. However, the scheduled specialist consultation was postponed and, at the time of manuscript revision, had not yet taken place. Consequently, no additional vascular imaging, venography, or interventional treatment had been performed, and no post-treatment clinical outcomes were available.

## Discussion

This case illustrates the diagnostic complexity of pelvic venous disease in patients with chronic pelvic pain and highlights several clinically relevant features that may contribute to its under-recognition. The patient presented with a symptom pattern compatible with pelvic venous disease, including persistent pressure-like pelvic pain aggravated by prolonged standing and sexual intercourse, associated with radiating low back pain and lower-limb heaviness. These manifestations are consistent with those described in pelvic venous disorders, in which pelvic pain or heaviness may worsen with prolonged standing and may be accompanied by dyspareunia or post-coital pain [2,3]. The patient was not multiparous and had no visible vulvar or perineal varices. Although multiparity has been associated with pelvic venous disease, it is not a diagnostic requirement, and the absence of these features does not exclude the condition [4].

The six-year interval between symptom onset and recognition of a possible pelvic venous etiology is particularly relevant. Chronic pelvic pain has a broad differential diagnosis encompassing gynecological, gastrointestinal, urological, musculoskeletal, neurological, and vascular conditions, and considerable symptom overlap may complicate recognition of a venous origin [3,5]. In this patient, symptoms had previously been attributed to recurrent gynecological infections and probable irritable bowel syndrome, while repeated assessments failed to establish a unifying diagnosis. This clinical trajectory is consistent with the challenges described in the literature, as pelvic venous disorders remain under-recognized and the absence of universally accepted diagnostic criteria may contribute to delayed diagnosis and repeated or inconclusive investigations [2,3]. The significance of this case, therefore, lies not in an unusual radiological manifestation, but in demonstrating how a possible vascular contributor to chronic pelvic pain may remain unrecognized for several years when symptoms are assessed separately rather than as part of a longitudinal clinical pattern.

Imaging findings should be interpreted in conjunction with the clinical presentation. The absence of abnormalities on an initial ultrasound does not exclude PCS, as diagnostic performance may depend on examination technique, patient positioning, Doppler assessment, evaluation during Valsalva maneuvers, and operator expertise [6,7]. A systematic review evaluating non-invasive diagnostic tools found promising diagnostic performance for ultrasonography and magnetic resonance imaging, although heterogeneity and methodological limitations prevented the establishment of definitive standardized criteria [6]. When clinical suspicion persists, further assessment may therefore include Doppler ultrasound, computed tomography (CT), or magnetic resonance imaging (MRI), while venography remains an important reference standard and may be performed in the context of planned endovascular treatment [2,4,6]. In the present case, non-contrast abdominopelvic CT demonstrated bilateral dilated periuterine veins measuring up to approximately 13 mm, an imaging finding that, in the clinical context, raised suspicion for pelvic venous disease.

Nevertheless, pelvic venous dilatation on imaging should not be considered diagnostic in isolation. Dilated or incompetent pelvic veins may also be identified in asymptomatic women, making clinical-radiological correlation essential when attributing chronic pelvic pain to pelvic venous disease [5]. This distinction is particularly important in PCS, where both symptoms and imaging findings may lack specificity. In the present case, the combination of positional and intercourse-related pain, lower-limb heaviness, and periuterine venous dilatation supported the clinical suspicion of pelvic venous disease, while not establishing a diagnosis on imaging alone.

Another noteworthy feature was the absence of vulvar or perineal varices on physical examination. Although superficial vulvar and perineal varices are recognized manifestations of pelvic venous disorders, they are not universally present [1-4]. Pelvic venous disease encompasses a heterogeneous spectrum of manifestations depending on the affected venous territories and underlying patterns of reflux or obstruction [3,4]. Consequently, the absence of externally visible varicosities should not prevent further investigation when the clinical presentation is otherwise suggestive. This case reinforces the importance of considering a pelvic venous origin even when superficial signs of venous insufficiency are absent.

Recognition of PCS is clinically relevant because potentially effective therapeutic options are available. Management should be individualized based on symptoms, venous anatomy, and patient preferences, and may include conservative, medical, endovascular, or surgical approaches [1,4]. Endovascular embolization of incompetent ovarian and pelvic veins has become an important minimally invasive treatment option in appropriately selected symptomatic patients [3,4]. A 2024 systematic review including 25 studies and 2,038 patients reported a technical success rate of 94%. Clinical outcomes were assessed separately, with studies reporting pre- and post-procedural pain scores generally demonstrating improvement following embolization. However, technical success should not be interpreted as equivalent to clinical improvement, and the authors highlighted substantial heterogeneity and the overall low quality of the available evidence [8,9]. In the present case, the patient was referred to vascular surgery for further assessment; however, the scheduled specialist consultation was postponed and had not yet taken place at the time of manuscript revision. Therefore, no decision regarding embolization had yet been made.

From a primary care perspective, this case highlights the value of continuity and longitudinal integration of clinical information. General and family medicine is often the setting in which recurrent symptoms, previous investigations, and assessments performed across different healthcare settings can be reviewed together. Recent literature directed specifically at family physicians has similarly emphasized the importance of recognizing pelvic venous disorders as an overlooked cause of chronic pelvic pain and initiating appropriate investigation and referral [8,10]. In this patient, integration of chronic pelvic pressure, positional worsening, dyspareunia, low back pain, and lower-limb heaviness prompted targeted imaging despite previous non-diagnostic assessments. Greater awareness of PCS in primary care may therefore facilitate earlier consideration of a pelvic venous cause in women with otherwise unexplained chronic pelvic pain, potentially reducing fragmented investigation and facilitating timely referral.

## Conclusions

Pelvic venous disease should be considered in the differential diagnosis of chronic pelvic pain when symptoms and imaging findings are suggestive, while recognizing that neither is specific in isolation. This case highlights the value of longitudinal assessment in general and family medicine for integrating persistent symptoms and previous investigations and facilitating appropriate specialist referral. In this patient, the clinical pattern and CT findings raised suspicion for pelvic venous disease; however, definitive specialist assessment remains pending.

## References

[1] Allaire C, Yong PJ, Bajzak K (2024). Guideline No. 445: management of chronic pelvic pain. J Obstet Gynaecol Can.

[2] Alsannan B, Alzeeny A, Etrusco A, Laganà AS, D'Amato A, Tulandi T (2024). Diagnosis and management of pelvic congestion syndrome: comprehensive review. Gynecol Obstet Invest.

[3] Clark MR, Taylor AC (2023). Pelvic venous disorders: an update in terminology, diagnosis, and treatment. Semin Intervent Radiol.

[4] Marcelin C, Le Bras Y, Molina Andreo I, Jambon E, Grenier N (2022). Diagnosis and management of pelvic venous disorders in females. Diagnostics.

[5] Borghi C, Dell'Atti L (2016). Pelvic congestion syndrome: the current state of the literature. Arch Gynecol Obstet.

[6] Steenbeek MP, van der Vleuten CJ, Schultze Kool LJ, Nieboer TE (2018). Noninvasive diagnostic tools for pelvic congestion syndrome: a systematic review. Acta Obstet Gynecol Scand.

[7] Brown CL, Rizer M, Alexander R, Sharpe EE 3rd, Rochon PJ (2018). Pelvic congestion syndrome: systematic review of treatment success. Semin Intervent Radiol.

[8] Brown AD (2025). Approach to pelvic venous disorders: primer for family physicians. Can Fam Physician.

[9] Hanna J, Bruinsma J, Temperley HC (2024). Efficacy of embolotherapy for the treatment of pelvic congestion syndrome: a systematic review. Ir J Med Sci.

[10] Knuttinen MG, Machan L, Khilnani NM, Louie M, Caridi TM, Gupta R, Winokur RS (2023). Diagnosis and management of pelvic venous disorders: AJR expert panel narrative review. AJR Am J Roentgenol.

